# Two new species of socially parasitic *Nylanderia* ants from the southeastern United States

**DOI:** 10.3897/zookeys.921.46921

**Published:** 2020-03-24

**Authors:** Steven J. Messer, Stefan P. Cover, Christian Rabeling

**Affiliations:** 1 School of Life Sciences, Arizona State University, Tempe, AZ 85287, USA Arizona State University Tempe United States of America; 2 Department of Entomology, Museum of Comparative Zoology, Harvard University, Cambridge MA 02138, USA Harvard University Cambridge United States of America

**Keywords:** Formicidae, inquiline syndrome, inquilinism, myrmecosymbiosis, social parasitism

## Abstract

In ants, social parasitism is an umbrella term describing a variety of life-history strategies, where a parasitic species depends entirely on a free-living species, for part of or its entire life-cycle, for either colony founding, survival, and/or reproduction. The highly specialized inquiline social parasites are fully dependent on their hosts for their entire lifecycles. Most inquiline species are tolerant of the host queen in the parasitized colony, forgo producing a worker caste, and invest solely in the production of sexual offspring. In general, inquilines are rare, and their geographic distribution is limited, making it difficult to study them. Inquiline populations appear to be small, cryptic, and they are perhaps ephemeral. Thus, information about their natural history is often fragmentary or non-existent but is necessary for understanding the socially parasitic life history syndrome in more detail. Here, we describe two new species of inquiline social parasites, *Nylanderia deyrupi***sp. nov.** and *Nylanderia parasitica***sp. nov.**, from the southeastern United States, parasitizing *Nylanderia wojciki* and *Nylanderia faisonensis*, respectively. The formicine genus *Nylanderia* is large and globally distributed, but until the recent description of *Nylanderia deceptrix*, social parasites were unknown from this genus. In addition to describing the new social parasite species, we summarize the fragmentary information known about their biology, present a key to both the queens and the males of the *Nylanderia* social parasites, and discuss the morphology of the social parasites in the context of the inquiline syndrome.

## Introduction

Ant social parasites exploit the social colony structure of free-living ant species, and they rely on their hosts for colony founding, survival, and reproduction for at least a part, and frequently the entirety of their life-cycles (Hölldobler and Wilson 1990; Buschinger 2009). Social parasitism is a life history strategy exhibited by at least 300 species among the approximately 13,500 described extant ant species. Traditionally, three main types of social parasitism have been recognized: temporary, dulotic, and inquiline social parasitism (Wasmann 1891; Wheeler 1910; Bourke and Franks 1991; Buschinger 2009). Inquilinism has evolved independently many times in the ants, and approximately 100 species are known from at least 30 ant genera which are distributed across six different subfamilies of the formicoid clade. Most inquilines do not produce a worker caste, and instead they invest their reproductive effort in producing sexual offspring. Many inquilines have convergently evolved a suite of similar morphological characteristics known as the “inquiline syndrome” (*sensu*Kutter 1968; Wilson 1971). These shared characteristics often include: elongated antennal scapes, reduced mouthparts, reduced body size, smooth and shiny cuticle, reduction or absence of the worker caste, intranidal mating with close relatives (i.e., adelphogamy), and polygyny (Kutter 1968; Wilson 1971, 1984; Hölldobler and Wilson 1990; Radchenko and Elmes 2003; Rabeling and Bacci 2010; Rabeling et al. 2015, 2019; Bharti et al. 2016). Interestingly, independently evolved inquiline species exhibit a mosaic of inquiline syndrome characteristics, frequently converging on a similar albeit not identical parasitic phenotype (Wilson 1984; Radchenko and Elmes 2003; Rabeling and Bacci 2010; Rabeling et al. 2019).

The genus *Nylanderia* is a member of the ant tribe Lasiini in the subfamily Formicinae (Blaimer et al. 2015) and presently consists of 150 described taxa (Bolton 2019; LaPolla and Kallal 2019). The genus is globally distributed, with the majority of species being found in warm, forested regions, though it is largely absent from the temperate regions of the Palearctic (LaPolla et al. 2011; Bolton 2019; LaPolla and Kallal 2019). In the Nearctic region, 14 native and six introduced species are recognized (LaPolla et al. 2010; Kallal and LaPolla 2012), representing a rather modest fauna given the high diversity and large biogeographic extent of the genus. Until recently, social parasitism was unknown in *Nylanderia* ants, and the first inquiline social parasite in the genus, *Nylanderia deceptrix* (Messer et al. 2016), was described from Massachusetts.

Here, we describe two new *Nylanderia* inquiline social parasites from the Nearctic and provide keys for identifying them. In addition, we summarize our current knowledge about the biology and natural history of these social parasites, and we briefly discuss the species morphologies and life histories in the context of the inquiline syndrome as well as inquiline evolutionary biology.

## Materials and methods

### Material examined

**ABS**Archbold Biological Station, Venus, FL, USA;

**MCZC**Museum of Comparative Zoology Collections, Harvard University, Cambridge, MA, USA;

**SIBR**Social Insect Biodiversity Repository, Arizona State University, Tempe, AZ, USA;

**USNM**National Museum of Natural History, Washington, DC, USA.

### Morphological analysis

Specimens were measured at the MCZ using a Wild M5A stereomicroscope (100× magnification) fitted with an ocular micrometer. Measurements were recorded and rounded to the nearest 0.01 mm at the highest magnification possible for each measurement and specimen. Composite images were generated at ASU using a Leica DFC450 digital camera mounted to a Leica M205 C stereomicroscope and assembled using Leica Application Suite (Version 4.5) and Helicon Focus (Version 6.6.1) software packages. Measurement terminology, abbreviations, and definitions follow LaPolla et al. (2011) and Kallal and LaPolla (2012):

**EL** (Eye Length): maximum length of compound eye in full-face view;

**GL** (Gaster Length): the length of the gaster in lateral view from the anteriormost point of the first gastral segment (third abdominal segment) to the posteriormost point (in males this included through the posterior end of parameres);

**HL** (Head Length): the length of the head proper, excluding the mandibles; measured in full-face view from the midpoint of the anterior clypeal margin to a line drawn across the posterior margin from its highest points (to accommodate species where the posterior margin is concave);

**HW** (Head Width): the maximum width of the head in full-face view (in males, portion of the eyes that extends past the lateral margins of the head is included);

**MMC** (Mesonotal Macrosetae Count): the number of erect macrosetae on mesonotum to one side of sagittal plane;

**MtMC** (Metanotal Macrosetae Count): the number of erect macrosetae on metanotum to one side of sagittal plane;

**MW** (Mesonotal Width): the maximum width of the mesonotum in dorsal view;

**PW** (Pronotal Width): the maximum width of the pronotum in dorsal view;

**PDH** (Propodeum Height): height of the propodeum as measured in lateral view from the base of the metapleuron to the maximum height of the propodeum;

**PFL** (Profemur Length): the length of the profemur from its margin with the trochanter to its margin with the tibia;

**PFW** (Profemur Width): the maximum width of the profemur;

**PL** (Paramere Length): the maximum length of the paramere;

**PMC** (Pronotal Macrosetal Count): the number of erect macrosetae on pronotum to one side of sagittal plane;

**SL** (Scape Length): the maximum length of the antennal scape excluding the condylar bulb;

**SMC** (Scape Macrosetal Count): the number of erect macrosetae on the scape visible in full frontal view;

**TL** (Total Length): HL+WL+GL;

**WL** (Weber’s Length): in lateral view, the distance from the posteriormost border of the metapleural lobe to the anteriormost border of the pronotum, excluding the neck;

**CI** (Cephalic Index): (HW/HL) × 100;

**FI** (Profemur Index): (FW/FL) × 100;

**REL** (Relative Eye Index): (EL/HL) × 100;

**SI** (Scape Index): (SL/HW) × 100.

### Statistical analysis of morphological measurements

To quantify morphological differences characteristic of the inquiline syndrome in *Nylanderia* ants, we collected morphological measurements for social parasites and their hosts and analyzed them statistically. We measured Weber’s Length (WL) as a proxy for Total Length (TL) because the gaster of individuals was often damaged during collection. Statistical analyses were conducted using R 3.4.0 (R Core Team 2017) statistical package. Due to low sample sizes that likely contributed to the data being non-normally distributed, we used Kruskal-Wallis tests to determine whether hosts and parasites were significantly different in size. In addition, we applied pairwise Mann-Whitney tests post-hoc to determine individual differences between social parasites and their respective hosts, as well as between the social parasites. A Bonferroni correction was applied to the Mann-Whitney tests to account for multiple comparisons between species and castes, and to provide a more conservative alpha to compensate for low sample sizes in some cases (see below). For the three *Nylanderia* host parasite pairs, we analyzed the following samples: *N. deyrupi*: 29 queens and 5 males; *N. parasitica*: 7 queens and 10 males; *N. deceptrix*: 22 queens and 5 males; *N. parvula* (Mayr, 1870): 19 queens, 13 males, and 15 workers; *N. wojciki* (Trager, 1984): 17 queens, 8 males, and 20 workers; and *N. faisonensis* (Forel, 1922): 17 queens, 10 males, and 29 workers. Morphological measures of *N. deceptrix* were taken during an earlier study (Messer et al. 2016), and we added the morphological measurements of free-living species reported by Kallal and LaPolla (2010) to our dataset.

Morphological examination revealed that the relative forewing length of social parasites is reduced in comparison to the hosts, therefore we measured wing length for *N. deceptrix* (*N* = 22), *N. deyrupi* (*N* = 20), and *N. parasitica* (*N* = 6) individuals and compared them to 128 individuals of 13 non-parasitic *Nylanderia* species, including: *N. arenivaga* (Wheeler, 1905) (*N* = 9), *N. austroccidua* (Trager, 1984) (*N* = 3), *N. bruesii* (Wheeler, 1903) (*N* = 1), *N. concinna* (Trager, 1984) (*N* = 14), *N. faisonensis* (*N* = 12), *N. hystrix* (Trager, 1984) (*N* = 1), *N. magnella* (Kallal & LaPolla, 2012) (*N* = 1), *N. parvula* (*N* = 30), *N. phantasma* (Trager, 1984) (*N* = 4), *N. querna* (Kallal & LaPolla, 2012) (*N* = 6), *N. terricola* (Buckley, 1866) (*N* = 11), *N. vividula* (Nylander, 1846) (*N* = 23), *N. wojciki* (*N* = 13). We calculated the Forewing Index (FWI), which is the ratio of Forewing Length to Weber's Length, to identify the relative wing size of each species. A Kruskal-Wallis test was used to determine if any significant difference in relative wing size was present. Pairwise Mann-Whitney tests, with a Bonferroni corrections, were used to identify significant differences between hosts and parasites. The same analyses were conducted with males of the following species of non-parasitic *Nylanderia* (*N* = 97): *N. arenivaga* (*N* = 5), *N. austroccidua* (*N* = 3), *N. bruesii* (*N* = 19), *N. concinna* (*N* = 12), *N. faisonensis* (*N* = 4), *N .hystrix* (*N* = 4), *N. magnella* (*N* = 3), *N. parvula* (*N* = 15), *N. phantasma* (*N* = 4), *N. querna* (*N* = 4), *N. terricola* (*N* = 12), *N. vividula* (*N* = 8), and *N. wojciki* (*N* = 4). We used all *N. parasitica* males (*N* = 10) in the analyses, but males of *N. deceptrix* and *N. deyrupi* were not included due to the absence of fully formed wings.

## Results

### Key to the males of Nearctic *Nylanderia* (modified from Kallal and LaPolla 2012)

**Table:** 

1	Antennae with 12 segments (Fig. 5A)	***N. parasitica* sp. nov.**
–	Antennae with 13 segments	**2**
2	Wings absent or highly reduced (Figs 1D, F; 3C, E)	**3**
–	Wings present and fully developed	start key from Kallal and LaPolla 2012
3	Gaster light brown in color similar to mesosoma, REL 27–28, SI 112–121 (Fig. 3C, E)	***N. deyrupi* sp. nov**.
–	Head and gaster dark brown in color contrasting with light brown mesosoma, REL 34–36, SI 125–127 (Fig. 1D, F)	***N. deceptrix***

### Key to the queens of Nearctic *Nylanderia*

**Table:** 

1	Scape Index (SI) < 113, Forewing Length > 2.4 mm, 6–7 mandibular teeth	**non-parasitic *Nylanderia***
–	SI ≥ 113, Forewing Length < 2.4 mm, < 6 mandibular teeth	**2**
2	Weber’s Length (WL) 0.99–1.07 mm; distinct bicoloration with darker head and gaster (Fig. 1A, C, E)	***N. deceptrix***
–	WL < 0.99 mm; head, mesosoma and gaster of uniform color	**3**
3	Mesonotal Macrosetae Count (MMC) 16–23, Metanotal Macrosetae Count (MtMC) 6–9, Pronotal Macrosetal Count (PMC) 7–11, and Scape Macrosetae Count (SMC) = 0, mandibular dentition absent (Fig. 4A, C, E)	***N. parasitica***
–	MMC 10–17, MtMC 2–4, PMC 2–6, and SMC > 0, 3–4 mandibular teeth (Fig. 2A, C, E)	***N. deyrupi***

**Figure 1.**
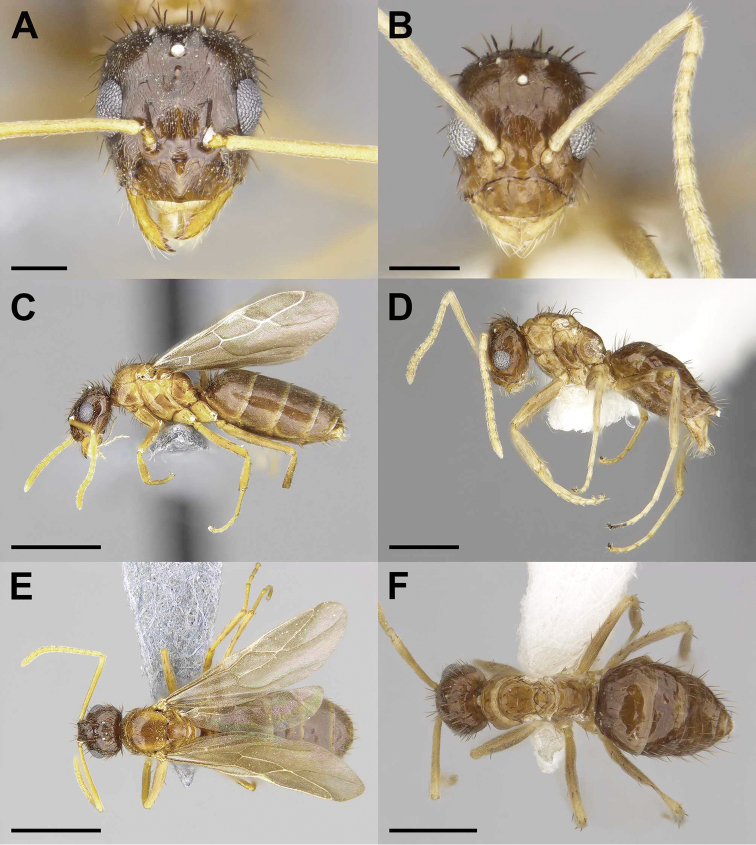
Gyne (**A, C, E**) and male (**B, D, F**) of the previously described social parasite *Nylanderia deceptrix* in full-face (**A, B**), lateral (**C, D**), and dorsal (**E, F**) views. Scale bars: 0.2 mm (**A, B)**, 1 mm (**C, E)**, 0.5 mm (**D, F).**

#### Nylanderia deyrupi sp. nov.

Taxon classificationAnimaliaHymenopteraFormicidae

1F47E322-1A96-563A-9948-D56C82796403

http://zoobank.org/7F338A9A-2545-4868-844D-D088510F7CDA

**Figure 2.**
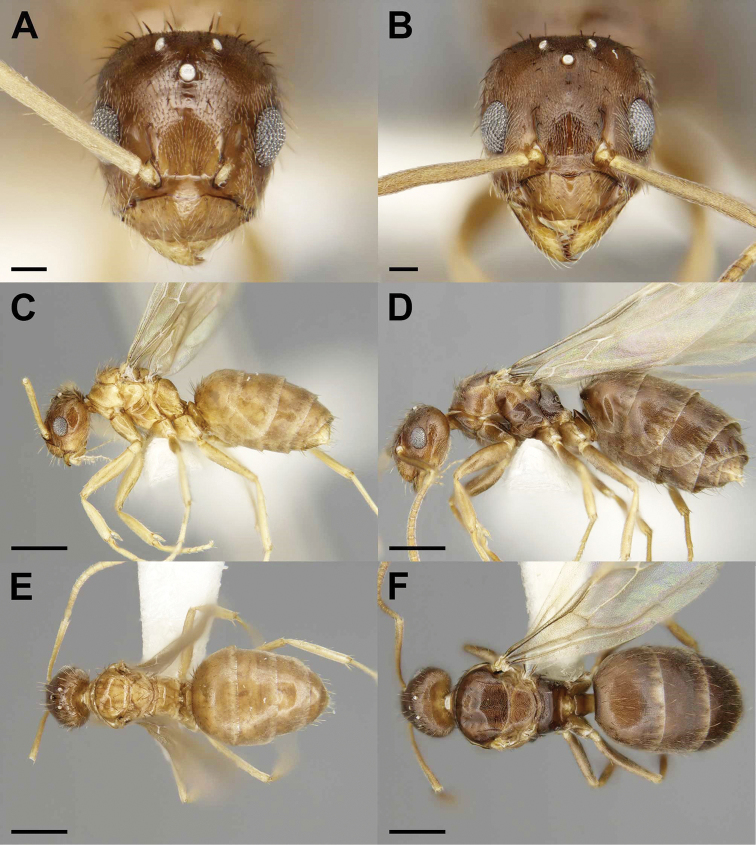
Gynes of the social parasite *Nylanderia deyrupi* (**A, C, E**) and its host *Nylanderia wojciki* (**B, D, F**) in full-face (**A, B**), lateral (**C, D**), and dorsal (**E, F**) views. Scale bars: 0.1 mm (**A, B**), 0.5 mm (**C–F**).

Figures 2A, C, E (queen), 3A, C, E (male); see Plate 88 in Deyrup (2016): p. 348.

##### Material examined.

***Holotype***: USA • alate queen; Florida, Highlands Co., Archbold Biological Station; 27.187N, 81.335W, elevation above sea level: 61 m; scrubby flatwoods, slash pine, *Quercus inopina*, *Q. geminata*, Palmetto, *Lyonia lucida*: under leaf-litter of oak canopy at edge gap in pure sand; 15-September-1995; Stefan P. Cover leg.; MCZ-ENT00716681. Deposited at MCZC.

***Paratypes***: USA • 1 alate queen; same data as for holotype; MCZ-ENT00716678 • 1 male; same data as for holotype; MCZ-ENT00716681 • 1 alate queen, 1 male (on same pin); same data as for holotype; MCZ-ENT00716684 • 1 alate queen, 1 male (on same pin); same data as for holotype; MCZ-ENT00716690 • 1 male; same data as for holotype; MCZ-ENT00716693 • 1 male; same data as for holotype; MCZ-ENT00716694. MCZ-ENT00716678, MCZ-ENT00716681, MCZ-ENT00716693, MCZ-ENT00716694 deposited at the MCZC; MCZ-ENT00716684, MCZ-ENT00716690 deposited at SIBR.

USA • 1 alate queen; Florida, Highlands Co., Archbold Biological Station; 27.187N, 81.335W, elevation above sea level: 61 m; malaise trap; 6-X-1983; Mark Deyrup leg.; ASUSIBR00000365 • 1 alate queen; same data as previous; but 8-X-1983; ASUSIBR00000366 • 1 alate queen; same data as previous; but 20-X-1983 ASUSIBR00000367 • 2 alate queens; same data as previous; but 26-X-1983; ASUSIBR00000368–369 • 3 alate queens; same data as previous; but 30-X-1983; ASUSIBR00000370–372 • 2 alate queens; same data as previous; but 15-XI-1983; ASUSIBR00000373–374 • 1 alate queen; same data as previous; but 19-XI-1983; ASUSIBR00000375 • 2 alate queens; same data as previous; but 23-IX-1985; ASUSIBR00000376–377 • 1 alate queen; same data as previous; but 4-X-1985; ASUSIBR00000378 • 1 alate queen; same data as previous; but 9-X-1985; ASUSIBR00000379 • 1 alate queen; same data as previous; but 12-X-1985; ASUSIBR00000380 • 1 alate queen; same data as previous; but 25-XI-1986; ASUSIBR00000381. ASUSIBR00000365-368, ASUSIBR000370-371, ASUSIBR00000373, ASUSIBR00000375-376, ASUSIBR00000378-381 deposited at MCZC; ASUSIBR00000369, ASUSIBR00000372, ASUSIBR00000374, ASUSIBR00000377 deposited at SIBR.

USA • 3 alate queens; Florida, Highlands Co., Archbold Biological Station; 27.187N, 81.335W; 25-Sept-2010; John LaPolla leg.; JSL100925-1/ASUSIBR00000382–384 • 1 alate queen; same data as previous; JSL100925-2/ASUSIBR00000385 • 1 alate queen; same data as previous; JSL100925-3/ASUSIBR00000386 • 3 alate queens; same data as previous; JSL100925-4/ASUSIBR00000387–389. ASUSIBR00000382, ASUSIBR00000389 deposited at MCZC; ASUSIBR00000383, ASUSIBR00000388 deposited at USMN; ASUSIBR00000384–387 deposited at SIBR.

**Figure 3.**
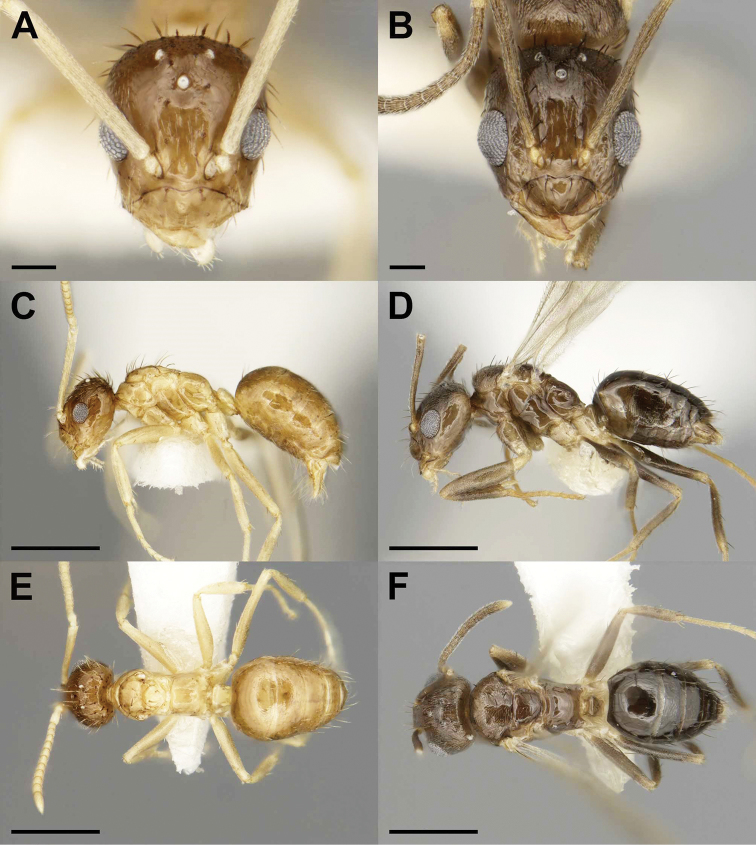
Males of the social parasite *Nylanderia deyrupi* (**A, C, E**) and its host *Nylanderia wojciki* (**B, D, F**) in full-face (**A, B**), lateral (**C, D**), and dorsal (**E, F**) views. Scale bars: 0.1 mm (**A, B**), 0.5 mm (**C–F**).

##### Diagnosis.

A workerless inquiline characterized by small alate queens and apterous males. Queens are easily distinguished from host queens by their smaller size (WL: *N. deyrupi* = 0.79–0.90 vs. *N. wojciki* = 1.10–1.16; Fig. 6), uniformly lighter coloration, long antennal scapes (SI = 118–130 vs. 86–101 in the host), reduced CI (86–94 vs. 95–97 in the host), reduced mandibular dentition (mandible = 3–4 teeth, host = 6 teeth), and reduced macrosetae counts on antennal scapes (2–6 vs. 1–2). In addition, the eyes exceed the lateral margins of the head and hind wing venation is slightly reduced. Males of *N. deyrupi* are completely apterous and are bicolored with a darker head and gaster and lighter mesosoma. The mesonotum is also reduced and narrow from the reduction of flight musculature and does not protrude anteriorly beyond the pronotum. Host males are fully alate and uniform brown. The antennal scapes lack macrosetae. Reduced REL (27–28 vs. 35–40) and SI increased (112–121 vs. 104–107).

Queens of *Nylanderia deyrupi* differ from those of the closely similar *N. deceptrix* by their smaller overall size (WL: *N. deyrupi* = 0.79–0.90 vs. *N. deceptrix* = 0.99–1.07; Fig. 7), reduced number of macrosetae on the mesonotum (10–17 vs. 21–27), smaller eyes (REL 24–31 vs. 33–37), and uniform coloration (queens of *N. deceptrix* are bicolored). *Nylanderia deyrupi* males are smaller in size (WL 0.55–0.58 vs. 0.66–0.69), possess more macrosetae on the mesonotum (4–5 vs. 2), pronotal macrosetae are absent, smaller eyes (REL 27–28 vs. 34–36), and shorter antennal scapes (SI 112–121 vs. 125–127).

##### Description, holotype gyne.

***Measurements***: TL 2.68, HW 0.56, HL 0.59, EL 0.17, SL 0.65, MW 0.47, PW 0.57, WL 0.85, GL 1.23, PDH 0.32, PFL 0.69, PFW 0.15, SMC 4, PMC 3, MMC 14, MtMC 3. ***Indices***: CI 94, REL 29, SI 118, FI 21. Small size (TL 2.68), body yellow-brown in color, dorsum of head and gaster slightly darker. ***Head***: covered in pubescence and macrosetae, slightly longer than wide (CI 94), broadening posteriorly, eyes protruding beyond lateral margins of head, three ocelli present. Maxillary and labial palp formula 6:4, mandibular dentition reduced to apical and three pre-apical teeth. Antennal scapes long (SI 118), exceeding posterior margin of head by length of first three funicular segments, covered in appressed setae with four erect macrosetae. Antennae 12-segmented. ***Mesosoma***: dorsum covered with pubescence, largely absent on lateral portions of mesosoma, pronotum bearing three macrosetae, mesonotum bearing 14 macrosetae, metanotum bearing three macrosetae, macrosetae matching body color, macrosetae on metanotum displaying significant curvature towards midline of body. Forewings smaller in size, but not distinctly different from forewings of host, hindwings with slightly reduced venation relative to host. ***Metasoma***: gaster covered in pubescence with clusters of macrosetae at anterior portion of first gastric tergite and posteriorly around acidopore.

##### Measurements, paratype gynes

(*N* = 28): TL 2.35–2.88, HW 0.51–0.56, HL 0.58–0.62, EL 0.15– 0.19, SL 0.64–0.67, MW 0.42–0.54, PW 0.42–0.62, WL 0.79–0.90, GL 0.96–1.38, PDH 0.30–0.35, PFL 0.55–0.72, PFW 0.09–0.15, SMC 2–6, PMC 2–6, MMC 10–17, MtMC 2–4. ***Indices***: CI 86–94, REL 24–31, SI 118–130, FI 16–24.

##### Description, paratype males.

***Measurements*** (*N* = 5): TL 1.80–1.84, HW 0.41–0.42, HL 0.44–0.46, EL 0.12–0.13, SL 0.46–0.50, MW 0.22–0.25, PW 0.28–0.31, WL 0.55–0.58, GL 0.78–0.83, PDH 0.21–0.23, PFL 0.48–0.51, PFW 0.10–0.11, PL 0.20–0.21, SMC 0, PMC 0, MMC 4–5, MtMC 1–2. ***Indices***: CI 90–93, REL 27–28, SI 112–121, FI 20–23. Overall yellowish-brown, exhibiting bicoloration with head and gaster darker than mesosoma, yellow color in legs, antennae and mandibles, macrosetae color matching body segment. ***Head***: slightly longer than wide (CI 90–93), covered in pubescence and macrosetae, denser posteriorly and laterally, eyes protruding beyond lateral margins of head, three ocelli present. Maxillary and labial palp formula 6:4, mandibular dentition reduced to apical tooth only. Antennal scapes long (SI 112–121), exceeding posterior margin of head by length of funicular segments I–III, covered in pubescence and lacking erect macrosetae. Antennae 13-segmented. ***Mesosoma***: small, completely apterous, largely lacking any pubescence. Pronotum lacking macrosetae, mesonotum offset posteriorly from pronotum and rising abruptly possessing four or five macrosetae, metanotum bearing one or two macrosetae that curve towards midline of body. Legs lacking macrosetae. ***Metasoma***: petiole triangular with longer posterior face sparsely covered in pubescence, macrosetae present on anterior of first gastral tergite and posterior margins of tergites and sternites. ***Genitalia***: parameres narrowly triangular, densely covered in macrosetae, slight mesad curvature at posterior end, digiti narrow and tubular, cuspi broad anteriorly and narrow laterally at posterior end.

##### Etymology.

This species is named in honor of Mark Deyrup, who first discovered the miniature females of *N. deyrupi* in malaise trap samples. Mark Deyrup has been a resident naturalist at Archbold Biological Research Station in Central Florida since 1982, and he is a uniquely gifted natural historian who acquired a phenomenal knowledge about the biology of the ants of Florida. Mark recently synthesized his knowledge in the richly illustrated monograph on the Ants of Florida (Deyrup 2016). His meticulous studies of ant natural history and taxonomy have inspired students and colleagues alike for decades, and without Mark’s insightful studies the rich natural history of Florida would be much less explored.

##### Distribution and natural history.

*Nylanderia deyrupi* is a rare, apparently workerless inquiline social parasite occurring only in nests of its host, *Nylanderia wojciki*. It is similar in morphology, and apparently in life-history, to *Nylanderia deceptrix*, the inquiline parasite of *N. parvula*. Its host, *N. wojciki* is native to Florida and the adjacent southeastern states. It is a common ant in sandhill and pine flatwood communities. In contrast, *N. deyrupi* is presently known only from Archbold Biological Station and two areas east of Sebring in Highlands County, Florida (Fig. 9; see also Deyrup 2016), all of which are located on the Lake Wales Ridge in central Florida. This sand ridge is more than one million years old (Turner et al. 2006), and it is home to endemic plants and animals, all narrowly distributed on the ridge itself. Deyrup (2016) suggested that *N. deyrupi* (referred to as *Nylanderia* Species A in Deyrup 2016) may be another such endemic species.

The host, *N. wojciki*, makes small (< 300 workers), usually monogynous colonies nesting in leaf litter or sand, usually in partly or lightly shaded areas (Trager 1984). The nests are often diffuse in the summer months, consisting of multiple shallow chambers within an area of 1–2 square meters, containing workers, brood, and sometimes sexuals. Collections of *N. deyrupi* consisted of queens and males scattered among these small host nest pockets, a pattern extremely similar to that seen in *N. deceptrix* and its host *N. parvula*. A striking feature of its life history, also shared with *N. deceptrix*, is that, unlike many ant inquilines, *N. deyrupi* does not appear to suppress the development of host sexuals. In collections made by M. Deyrup and S. Cover both host and parasite sexuals were commonly found together in the host nest, along with host worker brood and callows, strongly suggesting that the host queen is retained, not eliminated in parasitized colonies, coexisting with the social parasite. Another interesting life-history trait shared with *N. deceptrix* is the production of apterous males, which is unique among *Nylanderia* ants. Accordingly, males have limited mobility and probably no dispersal capability, and mating is expected to occur in or around the host nest (i.e., adelphogamy). Considering the limited mobility and the small number of males present in each nest, inbreeding is expected to occur in *N. deyrupi*. In addition, *N. deyrupi* has a strongly female-biased sex ratio, a phenomenon that has been frequently observed among inquiline social parasites.

##### Worker caste.

*Nylanderia deyrupi* was repeatedly collected from nests of *N. wojciki* and workers of *N. deyrupi* were never encountered. Thus, it is likely that *N. deyrupi* is a workerless inquiline social parasite.

#### Nylanderia parasitica sp. nov.

Taxon classificationAnimaliaHymenopteraFormicidae

BDB7DA3F-2627-59FE-B909-5D38364078BC

http://zoobank.org/DA163361-99CB-47AC-915B-5319A8298A7C

**Figure 4.**
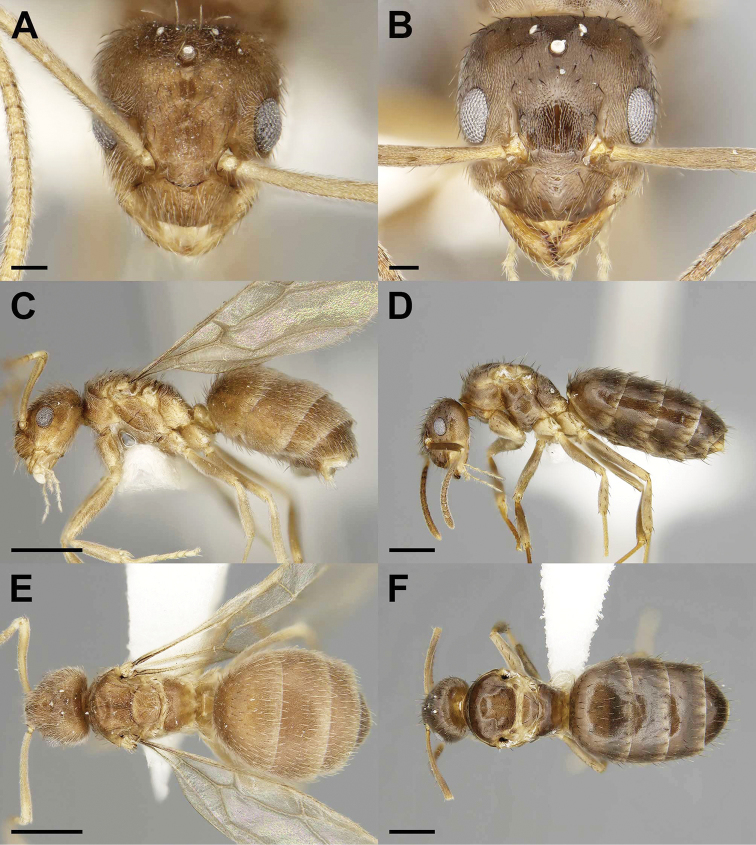
Gynes of the social parasite *Nylanderia parasitica* (**A, C, E**) and its host *Nylanderia faisonensis* (**B, D, F**) in full-face (**A, B**), lateral (**C, D**), and dorsal (**E, F**) views. Scale bars: 0.1 mm (**A, B**), 0.5 mm (**C–F**).

Figures 4A, C, E (queen), 5A, C, E (male); see Plate 89 in Deyrup (2016): p. 349.

##### Material examined.

***Holotype***: USA • alate queen; Florida, Hamilton Co., 2 miles east of Jasper, Route 6, pine-oak hammock near Snake Pond; 30.533N, 82.883W, elevation above sea level: 41 m; 03-July-1994; M. and S. Deyrup leg.; MCZ-ENT00716663. Deposited at MCZC.

***Paratypes***: USA • 7 males; same data as for holotype; MCZ-ENT00716664–666, MCZ-ENT00716668, MCZ-ENT00716670–672 • 1 alate queen, 1 male (on same pin); same data as for holotype; MCZ-ENT00716673 • 1 alate queen, 1 male (on same pin); same data as for holotype; MCZ-ENT00716674 • 1 alate queen, 1 male (on same pin); same data as for holotype; MCZ-ENT00716675. MCZ-ENT00716664–666, MCZ-ENT00716668, MCZ-ENT00716673 deposited at MCZC; MCZ-ENT00716670–672, MCZ-ENT00716674–675 deposited at SIBR.

USA • 1 alate queen; Florida, Alachua Co., Gainesville, Rock Creek; 9–17-IX-1983; S. Gupta leg.; MCZ-ENT00716676 • 1 alate queen; same data as previous; but V-1984; MCZ-ENT00716677. MCZ-ENT00716676 deposited at MCZC; MCZ-ENT00716677 deposited at SIBR.

USA • 1 alate queen; Georgia, Jones Co., Piedmont National Wildlife Refuge; 33.05N, 83.7167W; 19–26-VII-1994; J. Pickering leg.; MCZ-ENT00716662. Deposited at MCZC.

**Figure 5.**
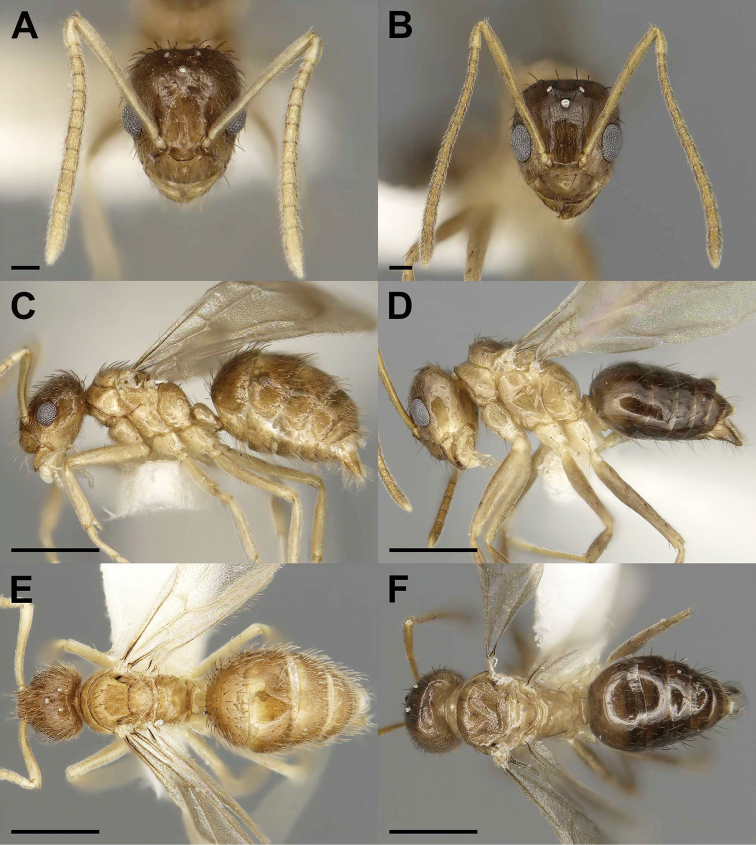
Males of the social parasite *Nylanderia parasitica* (**A, C, E**) and its host *Nylanderia faisonensis* (**B, D, F**) in full-face (**A, B**), lateral (**C, D**), and dorsal (**E, F**) views. Scale bars: 0.1 mm (**A, B**), 0.5 mm (**C–F**).

##### Diagnosis.

**Figure 6.**
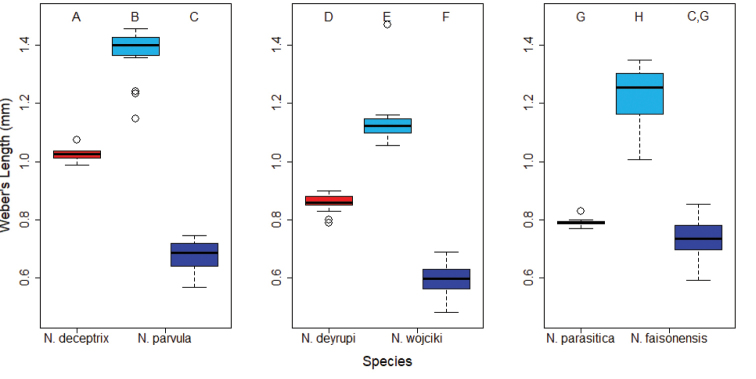
Boxplots comparing body sizes (Weber's Length) of social parasite queens (red) to the queens (light blue) and workers (dark blue) of their respective host species. Letters above the boxes indicate significantly different groups (Pairwise Mann-Whitney Test with Bonferroni correction, P < 0.05).

The queen of *N. parasitica* differs from the queen of its host, *N. faisonensis*, by its lightened coloration and smaller size (WL: *N. parasitica* = 0.77–0.83 vs. *N. faisonensis* = 1.00–1.35; Fig. 6). Macrosetae counts across the entire body of *N. parasitica* vs. *N. faisonensis* are higher: MMC (16–23 vs. 4–14), MtMC (6–9 vs. 1–3), and PMC (7–11 vs. 4–6); and macrosetae densely cover the whole gaster. Scape macrosetae are absent. The eyes also extend beyond the lateral margins of the head. Reduced CI (86–91 vs. 91–102), reduced REL (24–26 vs. 30–33), SI increased (113–119 vs. 102–112). Mandibular dentition reduced to an apical tooth and maximally two minute denticles as opposed to six mandibular teeth. Males are distinctly bicolored with a darker head and gaster, and the mesonotum is reduced and does not protrude beyond the pronotum. The pronotum possesses one or two macrosetae, which are absent in *N. faisonensis*. The antennae also have a reduced number of segments, possessing 12 as opposed to 13. CI increased (95–97 vs. 87–94) and reduced REL (26–29 vs. 34–36).

**Figure 7.**
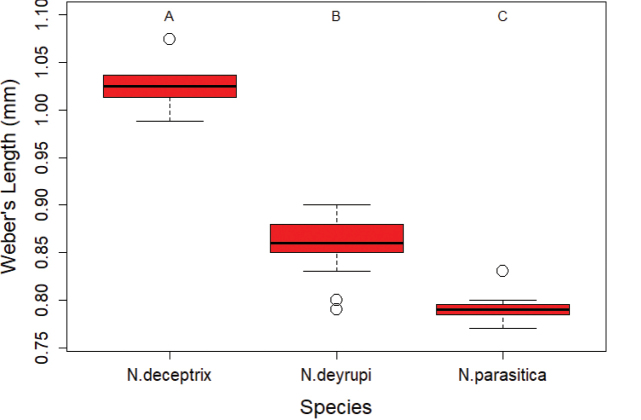
Boxplot comparing body sizes (Weber's Length) of social parasite queens to each other. Letters above the boxes indicate significantly different groups (Pairwise Mann-Whitney Test with Bonferroni correction, P < 0.05).

*Nylanderia parasitica* queens differ from those of *N. deceptrix* by: (i) possessing dense, pale macrosetae across the entire body, (ii) exhibiting a uniform body coloration, and (iii) an overall smaller size (WL: *N. parasitica* = 0.77–0.83 vs. *N. deceptrix* = 0.99–1.07; Fig. 7), (iv) the absence of macrosetae from the antennal scape, (v) reduced profemur size (FI 18–20 vs. 21–24), (vi) smaller relative eye size (REL 24–26 vs. 33–37), and (vii) shorter relative antennal scape length (SI 113–119 vs. 121–130). Mandibular dentition is reduced to an apical tooth and maximally two minute denticles vs. four or five mandibular teeth in *N. deceptrix*. In contrast to *N. deceptrix*, males of *N. parasitica* have (i) fully developed wings, (ii) pale macrosetae across the body, (iii) 1–2 pronotal macrosetae, (iv) a higher number of macrosetae on the metanotum (3–5 vs. 1–2), (v) macrosetae present on the gaster, (vi) a reduced FI (18–20 vs. 22–25), (vii) a reduced REL (26–29 vs. 34–36), (viii) a reduced SI (113–119 vs. 125–127), and (ix) 12-segmented antennae.

##### Description, holotype gyne.

***Measurements***: TL 2.54, HW 0.53, HL 0.59, EL 0.15, SL 0.60, MW 0.49, PW 0.56, WL 0.79, GL 1.16, PDH 0.31, PFL 0.67, PFW 0.14, SMC 0, PMC 9, MMC 16, MtMC 6. ***Indices***: CI 90, REL 26, SI 114, FI 20. *Nylanderia parasitica* is unique in the context of the Nearctic *Nylanderia* fauna because the queens are the smallest known to date (TL 2.54). ***Head***: slightly longer than wide (CI 90), broadening posteriorly, eyes protruding beyond lateral margins of head, three ocelli present. Maxillary and labial palp formula 6:4, mandibular dentition reduced to apical tooth and one small denticle. Antennae 12-segmented, scapes long (SI 114), exceeding posterior margin of head by length of first three funicular segments, covered in pubescence but lacking macrosetae. ***Mesosoma***: fully alate, pronotum bearing nine macrosetae, mesonotum bearing 16 macrosetae, metanotum bearing six macrosetae, mid and hind legs lacking macrosetae. Forewings showing no significant differences in venation from host, slight reduction in venation in hindwings. ***Metasoma***: lateral margins of petiole with pubescence and three macrosetae, gaster uniformly covered in dense pubescence and macrosetae. Body uniform yellow-brown in color with legs, antennae, and mandibles lighter yellow. All body regions densely covered in pale pubescence and macrosetae.

##### Measurements, paratype gynes

(*N* = 6): TL 2.27–2.54, HW 0.52–0.56, HL 0.58–0.61, EL 0.15, SL 0.60–0.63, MW 0.44–0.49, PW 0.49–0.57, WL 0.77–0.83, GL 0.89–1.16, PDH 0.30–0.32, PFL 0.60–0.67, PFW 0.11–0.14, SMC 0, PMC 7–11, MMC 16–23, MtMC 6–9. ***Indices***: CI 86–91, REL 24–26, SI 113–119, FI 18–20.

##### Description, paratype males.

***Measurements*** (*N* = 10): TL 1.70–2.10, HW 0.44–0.47, HL 0.46–0.49, EL 0.12–0.14, SL 0.51–0.54, MW 0.30–0.31, PW 0.36–0.38, WL 0.59–0.64, GL 0.62–0.99, PDH 0.22–0.25, PFL 0.52–0.56, PFW 0.10–0.11, PL 0.15–0.19, SMC 0, PMC 1–2, MMC 10–13, MtMC 3–5. ***Indices***: CI 95–97, REL 26–29, SI 113–119, FI 18–20. Body bicolored, pale yellow mesosoma with yellow-brown legs, head and gaster, head slightly darker than gaster, antennae and mandibles yellow. ***Head***: covered in pubescence and macrosetae, less dense than in female, slightly longer than wide (CI 95–97), eyes protruding beyond lateral margins of head, three ocelli present; maxillary and labial palp formula 6:4, mandibular dentition reduced to apical tooth only; antennal scapes long (SI 113–119), exceeding the posterior margin of head by length of funicular segments I–III, covered in pubescence, lacking erect macrosetae, antennae 12-segmented, reduced from typical 13-segmented in ants. ***Mesosoma***: dorsum covered in pubescence and macrosetae, largely absent on lateral portions of mesosoma, macrosetae matching body coloration, pronotum bearing one or two macrosetae, mesonotum bearing 10–13 macrosetae, metanotum bearing 3–5 macrosetae curving towards the midline of body; fully alate, wings resemble host with no significant differences. ***Metasoma***: petiole triangular with longer posterior face, 1–3 macrosetae present; gaster covered in pubescence and macrosetae, with macrosetae clustering on first gastral tergite and posterior margins of tergites and sternites. ***Genitalia***: parameres narrowly triangular, straight and densely covered in macrosetae, digiti narrow and tubular, cuspi broad anteriorly and narrow laterally at posterior end.

##### Etymology.

*Nylanderia parasitica* inhabits the nests of *N. faisonensis*, exhibits morphological characteristics of the inquiline syndrome, and potentially lacks a worker caste. Hence, the species epithet is indicative of the socially parasitic life history of *N. parasitica*.

##### Distribution and natural history.

Information on the natural history and biogeography of *N. parasitica* is extremely limited. In previous publications, *N. parasitica* was referred to as undescribed socially parasitic *Nylanderia* species (*N.* sp. 1) (Kallal and LaPolla 2012) and *Nylanderia* Species B (Deyrup 2016). Most individuals of *N. parasitica* were collected from Hamilton County, Florida. Two alate queens were collected from Alachua County, Florida, and a single alate queen was collected from Jones County, Georgia (Fig. 10; see also Deyrup 2016). The type series was collected inside the nest of *N. faisonensis* in a rotten log located in an upland oak-pine hammock and a pond swamp area in Hamilton County (Deyrup 2016). Unfortunately, no additional observations were recorded from this mixed colony. The two queens from Alachua County were collected in malaise traps in May and September, suggesting that *N. parasitica* females disperse on the wing throughout the warm summer months.

The host of *N. parasitica*, *N. faisonensis*, is widely distributed in the southeastern United States (Kallal and LaPolla 2012) and is primarily a woodland species living in rotting branches, under rotting tree bark, or in the leaf litter (Trager 1984). Nests are often fragmented, and they do not make a soil nest like most other Nearctic *Nylanderia* species, with the single queen living deep under the leaf litter, while workers and brood live near the surface (Trager 1984). Alates are reared from August to December, followed by an overwintering period typical of Nearctic *Nylanderia*. Alate individuals disperse from the maternal nests between March and May, while more southern populations start dispersing earlier during those months (Trager 1984). It remains unknown whether *N. parasitica* is tolerant of the *N. faisonensis* queen.

##### Worker caste.

Our limited collections of *N. parasitica* have not yielded any putative workers for this species. Therefore, like *N. deceptrix* and *N. parasitica*, it is likely that this species is a workerless inquiline.

### Morphometric analyses

**Body size.** Reduction of body size is a key characteristic of the inquiline syndrome, and to test the extent of body size reduction in *Nylanderia* social parasites, we compared social parasite queens and males to host queens, males, and workers. In general, queens of socially parasitic *Nylanderia* species were significantly different in body size when compared to host queens and workers (Kruskal-Wallis tests: *N. deceptrix* vs. *N. parvula*, χ^2^=37.39, df=2, P=7.6 × 10^-7^; *N. deyrupi* vs. *N. wojciki*, χ^2^ = 56.85, df = 2, P = 4.52 × 10^-12^; *N. parasitica* vs. *N. faisonensis*, χ^2^ = 37.3, df = 2, P = 7.94 × 10^-9^). Pairwise Mann-Whitney tests revealed that the inquiline queens were significantly smaller than their respective host queens (*N. deceptrix*, P = 5.0 × 10^-4^; *N. deyrupi*, P = 6.2 × 10^-7^; *N. parasitica*, P = 6.0 × 10^-3^) but larger than the host workers (*N. deceptrix*, P = 5.0 × 10^-4^; *N. deyrupi*, P = 6.2 × 10^-7^). *Nylanderia parasitica* marked the only exception where no significant size difference was detected between inquiline queens and host workers (P = 0.3; Fig. 6). Comparing the three social parasite queens to each other also revealed a significant size difference between the inquiline species (Kruskal-Wallis test: χ^2^ = 31.87, df = 2, P = 1.2 × 10^-7^; Fig. 7), with *N. deceptrix* being the largest and *N. parasitica* the smallest.

Comparing the social parasite males to the males of their respective host species, *N. deceptrix* and *N. deyrupi* were not significantly different in body size from the host males (Mann-Whitney tests: P = 0.44 and P = 1, respectively). In contrast, *N. parasitica* males were significantly smaller than *N. faisonensis* males (Mann-Whitney test: P = 0.01). When males of the three social parasite species were compared to each other, no significant difference in size was detected (Kruskal-Wallis test: χ2 = 2.67, df = 2, P = 0.26).

**Figure 8.**
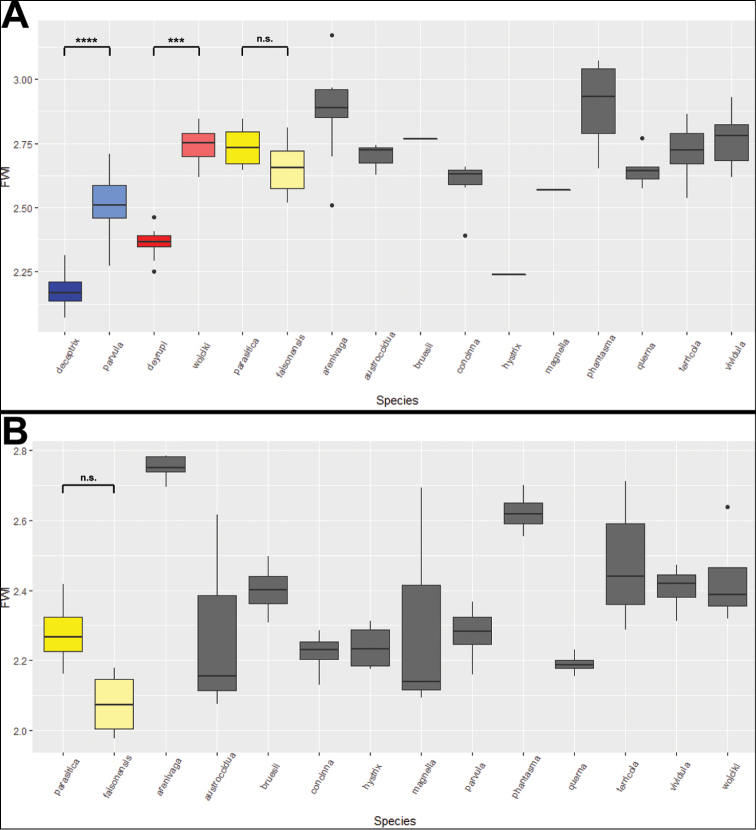
Boxplots of the Forewing Index (FWI) in (**A**) gynes of non-parasitic Nearctic *Nylanderia* (grey), *N. deceptrix* (red), *N. deyrupi* (blue), and *N. parasitica* (yellow), as well as (**B**) males of non-parasitic Nearctic *Nylanderia* (grey) and *N. parasitica* (yellow). Host species are represented by a lighter color shade than their respective social parasite species. (*** = P < 0.001, **** = P << 0.0001).

**Wing size.** Behavioral observations revealed that queens and males of *N. deceptrix* do not mate or disperse on the wing (Messer et al. 2016). Both inside nest mating and a reduced dispersal ability are important life history traits of inquiline social parasites, contributing to their localized distribution and frequently inbred population structure. Therefore, and as a proxy for flight performance, we measured the wings lengths of 13 free-living and three socially parasitic *Nylanderia* species. To test whether the social parasites have shorter relative wing lengths when compared to free-living *Nylanderia* species, we calculated the ratio of Forewing Length to Weber's Length and compared the values across Nearctic *Nylanderia* species for both queens and males. Significant differences between species were detected in both queens (Kruskal-Wallis test: χ2 = 140.46, df = 15, P < 2.2 × 10^-16^) and males (Kruskal-Wallis test: χ2 = 71.748, df = 13, P = 3.819 × 10^-10^). Pairwise Mann-Whitney tests determined that the wing sizes of *N. deceptrix* and *N. deyrupi* queens were significantly reduced relative to their host species (P = 2 × 10^-7^; P = 2.2 × 10^-4^, respectively; Fig. 8A). In contrast, both queens and males of *N. parasitica* did not exhibit any significant reduction in wing size relative to the host *N. faisonensis* (Mann-Whitney tests: P = 1; P = 0.364, respectively; Fig. 8B). Males of *N. deceptrix* and *N. deyrupi* were not included in the pairwise analysis, because they are brachypterous and apterous, respectively (Figs 1, 3).

## Discussion

We described two new workerless inquiline social parasite species in the genus *Nylanderia*, *N. deyrupi* and *N. parasitica*, from the southeastern United States. *Nylanderia deyrupi* was discovered in nests of *N. wojciki*, and *N. parasitica* was found once inside the nest of *N. faisonensis*. In ants, the presence of mixed colonies is indicative of a socially parasitic life history. *Nylanderia deyrupi* was collected repeatedly at or around Archbold Biological Station in central Florida, which yielded first insights into the biology of the species. In contrast, very little information is known about *N. parasitica*, which was only observed alive once in northern Florida. Therefore, our interpretations regarding the biology of the two species, especially of *N. parasitica*, should be regarded as preliminary and would greatly benefit from additional study. Notwithstanding, first observations suggest that *N. deyrupi* is a workerless, host queen tolerant inquiline because the *N. wojciki* queen, callow workers, and sexual brood were found inside the host colonies, whereas workers of *N. deyrupi* were absent. *Nylanderia parasitica* was only found in a mixed colony with *N. faisonensis*, and at the moment it remains unknown whether this inquiline species is host tolerant or not, but workers of *N. parasitica* were also absent from this mixed colony.

The description of these two social parasite species increases the diversity of Nearctic *Nylanderia* to 17 species, and three of them are inquiline social parasites. Approximately 100 species of inquiline social parasites are known from six ant subfamilies. The majority of the inquiline social parasites belong to the subfamily Myrmicinae, and only 12 inquiline species are known from the subfamily Formicinae, including the genera *Anoplolepis, Camponotus, Cataglyphis, Formica, Nylanderia, Plagiolepis*, and *Polyrhachis* (Hölldobler and Wilson 1990; Buschinger 2009; Karaman 2012; Casevitz-Weulersse 2014; Messer et al. 2016). Considering that inquiline social parasites are less common in formicine ants, these new *Nylanderia* inquiline species provide an opportunity for comparatively studying the morphological, behavioral, and ecological traits associated with inquiline social parasite evolution in formicine ants.

**Figure 9.**
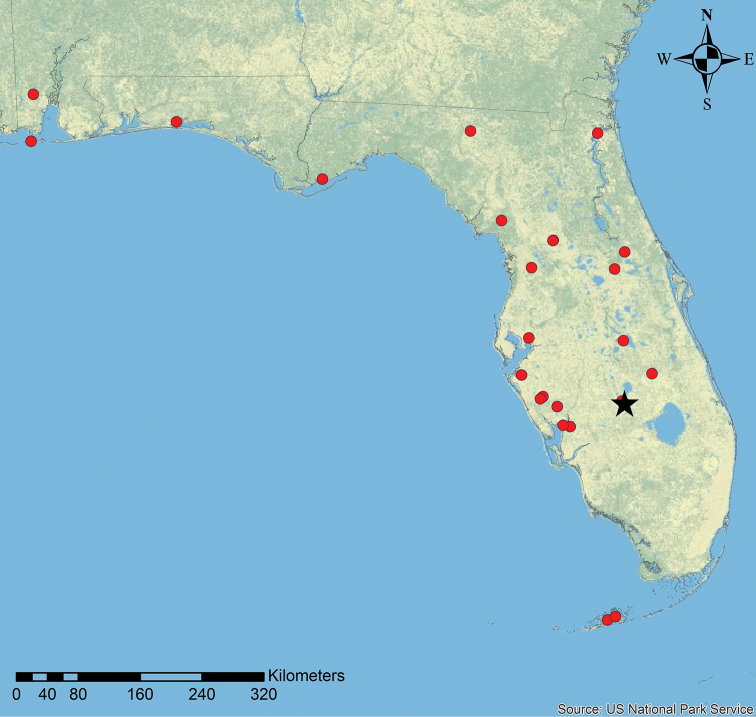
Geographic distribution of *N. deyrupi* (black star) and its host *N. wojciki* (red circles). Host distribution data was supplemented with additional information from antmaps.org (Janicki et al. 2016).

**Figure 10.**
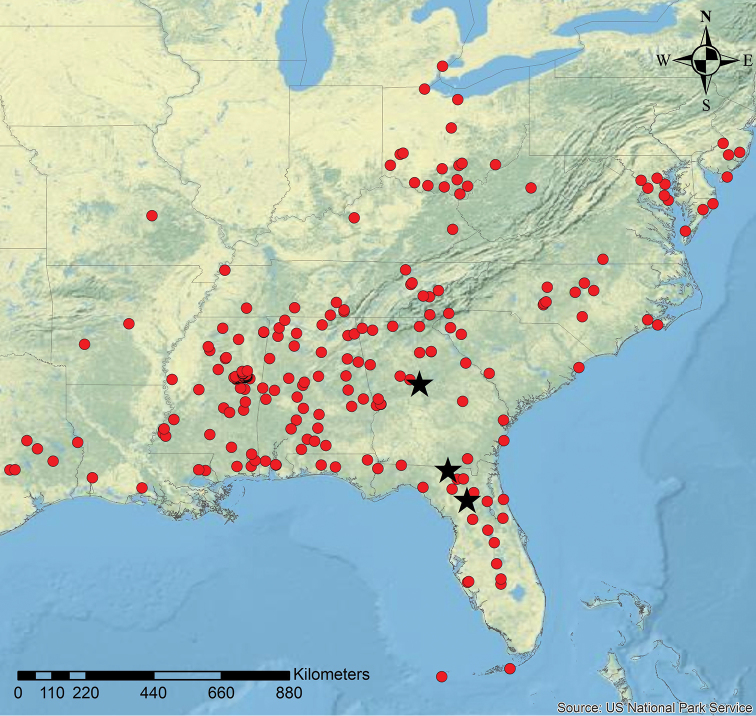
Geographic distribution of *N. parasitica* (black stars) and its host *N. faisonensis* (red circles). Host distribution data was supplemented with additional information from antmaps.org (Janicki et al. 2016).

*Nylanderia deyrupi* and *N. parasitica* seem to have limited geographic distribution ranges, which is typical for inquiline species (Wilson 1971; Buschinger 2009). So far, *N. deyrupi* is known from central Florida (Fig. 9), while *N. parasitica* was collected in northern Florida and southern Georgia (Fig. 10) (Deyrup 2016). These distribution ranges are significantly smaller than the ranges of their respective host species (Figs 9, 10) (Trager 1984; Kallal and LaPolla 2012; Deyrup 2016). However, *N. parasitica* has a considerably larger known range compared to both *N. deceptrix* and *N. deyrupi*. Considering that males of *N. parasitica* are fully winged and that queens were collected in Malaise traps, it is possible that mating and/or dispersal flights occur in this species, which could contribute to a wider geographic distribution. The currently recognized biogeographic distribution almost certainly also reflects sampling biases, considering that inquiline social parasites are rarely found.

### The inquiline syndrome of *Nylanderia* social parasites

Social parasites display a mosaic of morphological, behavioral, and life history traits characteristic of their socially parasitic biology, known as the inquiline syndrome (Kutter 1968; Wilson 1971). *Nylanderia* inquiline social parasites show adaptations and losses associated with a socially parasitic life history, including a loss of the worker caste, polygyny, elongated scapes, lighter coloration, reduced body sizes, reduced wings, and a loss of antennal segments (Table 1). Other inquiline syndrome characters outlined by Wilson (1971) and Hölldobler and Wilson (1990), such as reduced labial and/or maxillary palps, a smooth and shiny cuticle, and a reduced pilosity could not be observed in *Nylanderia* social parasites, supporting the hypothesis that morphological, behavioral, and life history traits characteristic of a socially parasitic lifestyle evolve convergently in a mosaic fashion (Wilson 1984; Hölldobler and Wilson 1990; Radchenko and Elmes 2003; Rabeling and Bacci 2010; Rabeling et al. 2015, 2019). We briefly discuss the most significant modifications observed in *Nylanderia* inquiline social parasites.

**Table 1.** Comparison of inquiline syndrome characteristics for *N. deceptrix, N. deyrupi*, and *N. parasitica*. Traits applying to females but not males are marked with an asterisk (*), whereas traits applying to males but not females are marked with a cross (^+^). Morphological reductions observed in social parasites were determined by comparisons relative to the respective host species.

	*N. deceptrix*	*N. deyrupi*	*N. parasitica*
Loss of worker caste	X	X	X
Presence of multiple parasite queens in host colony (polygyny)	X	X	X
Coexistence with host queen (host-queen tolerance)	X	X	?
Reduced body size	X	X	X
Limited geographic distribution	X	X	X
Reduced wing venation	X	X	–
Reduced mouthparts	–	–	–
Reduced antennal segments	–	–	X^+^
Smooth, shiny integument	–	–	–
Elongated scapes	X	X	X*
Reduced pilosity	–	–	–
Reduced wings	X	X	–
Reduced mandibular dentition	X	X	X

**Body size reduction.** In comparison to their hosts, all three *Nylanderia* social parasite species are significantly reduced in size. A comparative analysis of the inquiline syndrome in *Pheidole* and fungus-growing ant social parasites revealed that body size reduction is one of the first traits to evolve in inquilines (Wilson 1984; Rabeling and Bacci 2010). Nonacs and Tobin (1992) conducted an analysis of inquiline size relative to their hosts, using head size as a proxy for body size, and discovered that the queens of 18 of the 19 species in their study were equal in size or smaller than the host workers. A behavioral study of *Plagiolepis* inquilines examined the effect of size reduction on social parasite survival, revealing that miniaturization prevented *P. xene* queen and male brood from being culled by host workers (Aron et al. 1999, 2004). In contrast, *P. pygmaea* host males, which are larger than the host workers, were actively removed from the colony (Aron et al. 1999, 2004). In *Nylanderia*, only *N. parasitica* queens are similar in size to the host workers, which is consistent with the pattern observed by Nonacs and Tobin (1992). However, *N. deceptrix* and *N. deyrupi* show a lesser degree of size reduction with both inquiline species displaying intermediate sizes between their respective host queens and workers. It would be insightful to conduct behavioral experiments to test whether a lesser degree of body size reduction in these inquilines increases their risk of being detected and removed by the host.

**Wing size reduction.** Studying the wing morphology of social parasites is important for inferring the species mating and dispersal behavior. Both mating and dispersal behavior can be highly modified in social parasites, and many inquiline species are known to mate with their siblings inside the host nest instead of performing a mating flight, contributing to an inbred population structure and to a restricted biogeographic distribution of the species (Alpert and Akre 1973; Buschinger 1989; Bourke and Franks 1991; Aron et al. 1999; Buschinger and Linksvayer 2004; Trontti et al. 2005; Satoh and Ohkawara 2008; Rabeling and Bacci 2010; Heinze et al. 2015). Queens of *N. deceptrix* have reduced wings in comparison to the host and behavioral tests revealed the queens’ inability to fly (Messer et al. 2016). *Nylanderia deyrupi* also has significantly smaller wings relative to the host (Fig. 8A), and the males of both *N. deceptrix* and *N. deyrupi* are brachypterous and apterous, respectively, suggesting that both species likely mate inside or close to the host nest. Interestingly, alate queens of *N. deyrupi* were collected in malaise traps. If the trap was not installed on top of the nest and *N. deyrupi* queens did not simply crawl into the trap, this observation suggests that alates may mate in the host nest but that queens are still capable of dispersing on the wing. In contrast, both queens and males of *N. parasitica* do not have significantly smaller wings when compared to the host (Fig. 8A, B) and the wider geographic distribution could be indicative of mating and/or dispersal flights occurring in *N. parasitica*. Direct observations of the mating behavior are missing for all three social parasite species, however, and when sufficient samples become available, future studies need to test directly for population genetic signatures of inbreeding in *Nylanderia* social parasites.

**Reduction of antennal segments.** One trait of the inquiline syndrome that is unique to *N. parasitica* and absent from *N. deceptrix* and *N. deyrupi* is the reduction in the number of antennal segments from 13 to 12 in males. A reduction of antennal segments has been observed in some social parasite species of fungus-growing ants, such as *Pseudoatta argentina* and *Mycocepurus castrator* (Gallardo 1916; Rabeling and Bacci 2010). The reduction of antennal segments is potentially correlated with a reduced number of olfactory receptors, but this hypothesis remains to be tested.

### Outlook

With currently three known social parasite species, the genus *Nylanderia* developed into an interesting study system for exploring the evolutionary history of social parasitism in a comparative context. In general, inquiline social parasites are of interest to evolutionary biology because of their departures from a free-living life history, the convergent morphological and behavioral evolution of traits associated with the socially parasitic life history, as well as their close phylogenetic relationships to their hosts. Previous studies revealed that some inquiline species evolved directly from their host species via sympatric speciation (Savolainen and Vepsäläinen 2003; Rabeling et al. 2014; Leppänen et al. 2015; Nettel-Hernanz et al. 2015) whereas other inquilines likely originated in allopatry (Agosti 1994; Sanetra and Buschinger 2000; Ward et al. 2015). In a forthcoming study, we will test whether *Nylanderia* inquilines evolved via the intra- or the interspecific route of social parasite evolution. In addition to inferring the evolutionary history, it is critical to study the behavior and natural history of *Nylanderia* inquilines to gain a more detailed understanding of their biology.

## Supplementary Material

XML Treatment for Nylanderia deyrupi

XML Treatment for Nylanderia parasitica
